# Rapid Laboratory Identification of *Neisseria meningitidis* Serogroup C as the Cause of an Outbreak — Liberia, 2017

**DOI:** 10.15585/mmwr.mm6642a5

**Published:** 2017-10-27

**Authors:** Jaymin C. Patel, Josiah George, Jeni Vuong, Caelin C. Potts, Catherine Bozio, Thomas A. Clark, Jerry Thomas, Joshua Schier, Arthur Chang, Jessica L. Waller, Maureen H. Diaz, Melissa Whaley, Laurel T. Jenkins, Serena Fuller, Desmond E. Williams, John T. Redd, Ray R. Arthur, Fahn Taweh, Yatta Vera Walker, Patrick Hardy, Maxwell Freeman, Victoria Katawera, Gulu Gwesa, Miatta Z. Gbanya, Peter Clement, Henry Kohar, Mardia Stone, Mosoka Fallah, Tolbert Nyenswah, Jonas M. Winchell, Xin Wang, Lucy A. McNamara, E. Kainne Dokubo, LeAnne M. Fox

**Affiliations:** ^1^Epidemic Intelligence Service, CDC; ^2^Divison of Bacterial Diseases, National Center of Immunization and Respiratory Diseases, CDC; ^3^National Public Health Institute of Liberia, ^4^Laboratory Leadership Service, CDC; ^5^Division of Reproductive Health, National Center for Chronic Disease Prevention and Health Promotion, CDC; ^6^Division of Laboratory Services, National Center for Environmental Health, CDC; ^7^Division of Environmental Hazards and Health Effects, National Center for Environmental Health, CDC; ^8^Division of Global Health Protection, Center for Global Health, CDC; ^9^World Health Organization, Monrovia, Liberia; ^10^Ministry of Health and Social Welfare of Liberia, Liberia.

On April 25, 2017, a cluster of unexplained illness and deaths among persons who had attended a funeral during April 21–22 was reported in Sinoe County, Liberia (*1*). Using a broad initial case definition, 31 cases were identified, including 13 (42%) deaths. Twenty-seven cases were from Sinoe County (*1*), and two cases each were from Grand Bassa and Monsterrado counties, respectively. On May 5, 2017, initial multipathogen testing of specimens from four fatal cases using the Taqman Array Card (TAC) assay identified *Neisseria meningitidis* in all specimens. Subsequent testing using direct real-time polymerase chain reaction (PCR) confirmed *N. meningitidis* in 14 (58%) of 24 patients with available specimens and identified *N. meningitidis* serogroup C (NmC) in 13 (54%) patients. *N. meningitidis* was detected in specimens from 11 of the 13 patients who died; no specimens were available from the other two fatal cases. On May 16, 2017, the National Public Health Institute of Liberia and the Ministry of Health of Liberia issued a press release confirming serogroup C meningococcal disease as the cause of this outbreak in Liberia.

Meningococcal disease, caused by the bacterium *N. meningitidis*, is a serious febrile illness that most commonly manifests as meningitis or septicemia. *N. meningitidis* is classified into 12 serogroups based on its polysaccharide capsule; however, six serogroups (A, B, C, W, X, and Y) are responsible for a majority of meningococcal disease cases worldwide.* Meningococcal meningitis is characterized by sudden onset of fever, headache, stiff neck, nausea, vomiting, photophobia, or confusion. Meningococcal septicemia often begins with nonspecific signs and symptoms such as fever, vomiting, and diarrhea; in later stages, a hemorrhagic purpuric rash often occurs. However, absence of fever as well as hypothermia have been reported among persons with severe meningococcal septicemia (*2*,*3*). Worldwide, the greatest burden of meningococcal disease is in the African meningitis belt, which stretches from Senegal to Ethiopia, but does not include Liberia. Historically, *N. meningitidis* serogroup A (NmA) was responsible for majority of the epidemics in the meningitis belt. However, since 2010, the phased introduction of a meningococcal serogroup A conjugate vaccine (PsA–TT, MenAfriVac) throughout the meningitis belt, has substantially reduced NmA incidence and eliminated NmA epidemics (*4*). Recently, the region has experienced epidemics of NmC occurring in Niger (2015, 2017) (*5*) and Nigeria (2017).^†^

The cases of unexplained illness in Liberia were tightly clustered in time, with illness onset from April 21, 2017, through April 30, 2017 (Figure). The outbreak case definition comprised two or more symptoms including headache, vomiting, mental confusion, or weakness, with illness onset on or after April 10, 2017, in any person who visited or lived in Sinoe County (*1*). Among the 31 reported cases, the predominant reported signs and symptoms included weakness (28; 90%), abdominal pain (25; 81%), headache (24; 77%), and vomiting (20; 65%); fever was reported in only six (19%) patients. There were no reports of travel outside Liberia among the funeral attendees. Upon identification of the cluster, oral swab and blood specimens from patients were immediately tested in Liberia for Ebola virus and Lassa virus; both were ruled out. Because most of the patients were afebrile, a noninfectious etiology was considered likely; however, the nonspecific symptoms reported could have also been caused by an infection. Specimens collected from patients during the outbreak investigation were sent to multiple international laboratories for additional testing. On May 2, 2017, seven specimens (three whole blood, three oral swabs, and one plasma) from four fatal cases and three urine specimens from three nonfatal cases were received at CDC headquarters in Atlanta for testing. The urine specimens from the three nonfatal cases were tested for toxic metals and organophosphate insecticide metabolites; findings were not consistent with an exposure that could explain the outbreak. On May 5, 2017, six specimens (three whole blood, one plasma, and two oral swab specimens) from the four fatal cases were tested using the TAC assay. Developed at CDC, the TAC assay is a rapid diagnostic microfluidics-based real-time PCR assay that allows for simultaneous detection of approximately 40 viral, bacterial, and parasitic pathogens found in blood or cerebrospinal fluid (*6*). *N. meningitidis* was detected in all six specimens. On May 6, 2017, all specimens from the four fatal cases, including the six specimens tested by TAC assay and the additional oral swab specimen, underwent confirmatory testing by direct real-time PCR at CDC. Using a molecular target different from the one used in the TAC assay, *N. meningitidis* species was confirmed in all seven specimens and NmC was identified as the specific serogroup in all seven specimens.

**FIGURE:**
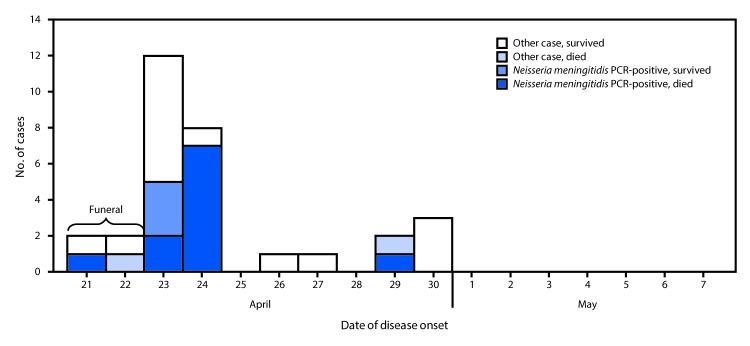
Date of onset of outbreak cases (N = 31), by laboratory and outcome status — Liberia, 2017* **Abbreviation:** PCR = polymerase chain reaction. * Other cases include PCR-negative and untested outbreak cases.

On May 9, 2017, CDC staff members deployed to Liberia to establish direct real-time PCR capacity for *N. meningitidis* testing in Liberia. Working with Liberian counterparts, the CDC team tested 56 additional specimens from 24 of the 31 cases initially identified as part of the outbreak. Overall, *N. meningitidis* was detected in specimens from 14 (58%) patients, 13 (54%) of which were confirmed as NmC and one which was nongroupable Nm (negative for invasive serogroups A, B, C, W, X, and Y). Notably, *N. meningitidis* was detected in specimens from 11 of the 13 patients who died; specimens from the other two fatal cases were not available for testing. On May 16, 2017, the National Public Health Institute of Liberia and the Ministry of Health of Liberia declared that the cluster of illness had been confirmed as a serogroup C meningococcal disease outbreak.

Patients who tested positive for *N. meningitidis* by PCR (14 patients) had more severe illness than did those who tested negative (10 patients). The interval from symptom onset to hospital admission was shorter among PCR-positive patients (median = 1 day, range = 0–2 days) than among PCR-negative patients (median = 6.5 days; range = 1–10 days), and 11 of the 14 PCR-positive patients died, whereas all 10 PCR-negative patients survived. Among the 11 PCR-positive patients who died, the median interval from symptom onset to death was 1 day (range = 0–4 days).

In addition to rapid testing of outbreak specimens in Liberia, the CDC team trained the Liberian National Public Health Reference Laboratory staff members in the use of direct real-time PCR and culture for the three main bacterial meningitis pathogens in sub-Saharan Africa, *N. meningitidis*, *Haemophilus influenzae*, and *Streptococcus pneumoniae.* The CDC team also provided training on transport and storage of specimens from patients with suspected meningitis. These trainings strengthened capacity for meningitis testing in Liberia.

## Discussion

In this outbreak, after ruling out Ebola and Lassa virus, the low prevalence of fever, high prevalence of gastrointestinal symptoms, and clustered onset of illness resulted in a broad differential diagnosis that initially focused on toxic exposures rather than infectious disease. In addition, because Liberia is not located within the African meningitis belt, there was not a high index of suspicion for meningococcal disease. The prompt identification of the cluster by the Liberian authorities and the rapid response from CDC, which included testing using the TAC assay and direct real-time PCR, allowed *N. meningitidis* to be identified as the cause of the outbreak. Because *N. meningitidis* often colonizes the nasopharynx asymptomatically and can be transmitted by asymptomatic carriers, it is not possible to ascertain how the outbreak strain of NmC was introduced into this population.^§^

The high prevalence of gastrointestinal symptoms among cases in this outbreak is unusual for a meningococcal disease cluster; however, a serogroup W meningococcal disease cluster with a high prevalence of gastrointestinal symptoms and a high case-fatality rate was recently reported from England (*7*). Generally, gastrointestinal symptoms are more commonly observed with meningococcal septicemia than with meningitis. The available data on meningococcemia in the African meningitis belt are sparse, but meningitis appears to be a more common clinical manifestation of *N. meningitidis* infection in this region than meningococcemia. Furthermore, in previous outbreaks, reported meningococcemia cases also presented with symptoms of meningitis (*8*,*9*). The high case-fatality rate is consistent with findings from other meningococcal disease outbreaks (*7**,**8*).

The use of a broad initial case definition was appropriate, given that the etiology of the outbreak was unknown; however, this case definition could capture patients with mild symptoms who are unlikely to have meningococcal disease. *N. meningitidis* was not detected in specimens from all patients, and the patients whose specimens tested negative by PCR had milder illness than did those who tested positive. This finding suggests that some PCR-negative patients likely did not have meningococcal disease; however, meningococcal disease cannot be ruled out definitively for patients who tested negative, because some specimens might have been collected several days after antibiotic treatment was initiated, when PCR might have lower sensitivity to detect *N. meningitidis (**10**)*.

This experience illustrates the importance of rapid laboratory confirmation in an outbreak investigation. The rapid detection of and response to the outbreak by Liberian health authorities is particularly noteworthy and highlights the impact of global health security capacity-building efforts on improving public health laboratory and emergency response capacities. This investigation also highlights the utility of the TAC assay in diagnosing outbreaks of unknown etiology, and shows the effectiveness of direct real-time PCR as a diagnostic tool for rapid response. Determination of the etiology of this outbreak enabled Liberian health authorities to implement appropriate response measures. With PCR capacity for identification of common causes of bacterial meningitis now available within the country, Liberia is in a stronger position to rapidly diagnose and effectively respond to additional meningococcal disease cases or outbreaks.

**Summary**

**What is already known about this topic?**

Meningococcal disease caused by the bacterium *Neisseria meningitidis* is a serious illness that commonly manifests as meningitis or septicemia. Globally, the highest disease burden is observed in the African meningitis belt that stretches from Senegal to Ethiopia; however, sporadic meningococcal disease cases and outbreaks also occur worldwide.

**What is added by this report?**

Following the detection of an outbreak of an unknown etiology surrounding a funeral event in Liberia, a rapid laboratory response using the Taqman Array Card (TAC) and confirmatory direct real-time polymerase chain reaction (PCR) assays identified *N. meningitidis* serogroup C as the cause of the outbreak.

**What are the implications for public health practice?**

Prompt and accurate detection of outbreaks allows public health officials to respond quickly and implement appropriate control measures. This report underscores the utility of TAC assay and direct real-time PCR in diagnosing outbreaks of unknown etiology.

## Footnotes

* http://www.who.int/csr/disease/meningococcal/en/.

† Nigeria Centre for Disease Control. Cerebrospinal Meningitis Outbreak in Nigeria. Situational Report, 2017. http://ncdc.gov.ng/themes/common/files/sitreps/bd9846806324bec6e408c3c4e696e63e.pdf.

§ http://www.sciencedirect.com/science/article/pii/S1473309910702516.
