# *Erratum*: Vol. 66, No. 42

**DOI:** 10.15585/mmwr.mm6645a7

**Published:** 2017-11-17

In the report “Rapid Laboratory Identification of *Neisseria meningitidis* Serogroup C as the Cause of an Outbreak — Liberia, 2017,” on page 1145, the figure title was not included in the printed version of this report. The figure title should have read “**FIGURE. Date of onset of outbreak cases (N = 31), by laboratory and outcome status — Liberia, 2017**.*”

